# Exploring health provider’s knowledge on the home-based maternal and neonatal health care package in Rwanda

**DOI:** 10.1186/s12884-022-04435-2

**Published:** 2022-02-07

**Authors:** Clemence Nishimwe, Gugu G. Mchunu

**Affiliations:** 1 School of Nursing and Public Health, University of KwaZulu-Natal, College of Health Sciences, King George Ave, Durban, 4001 South Africa; 2 Health Economics and HIV/AIDS Research Division (HEARD), University of KwaZulu-Natal, Durban, South Africa

**Keywords:** Health care provider, knowledge, home based, maternal and neonatal health care package, strategy, Rwanda

## Abstract

**Background:**

Rwanda implemented post-natal care home visits by maternal community health workers (M-CHWs) in charge of maternal and newborn health care in 2010 as a component of a home–based maternal and neonatal health care package (HB-MNHCP), this being a complementary strategy to facility-based postnatal care to improve survival. The country has not met its Sustainable Development Goal (SDG) 3 target of less than 70 maternal mortalities per 100,000 live births and less than 12 neonatal deaths per 1,000 live births. This study therefore aimed to establish the knowledge of the health providers, providing HB-MNHC services as part of their antenatal, delivery and postnatal care program, specifically the M-CHWs services.

**Methods:**

The cross-sectional descriptive study included 79 purposively sampled health care providers who were directly involved in the various components of the HB-MNHCP, namely: professional nurses, midwives, M-CHW, social workers, supervisors and data managers. The Kibogora, Muhima and Nyamata District Hospitals and two rural, semi-urban and urban health facility were included. Data was collected using questionnaires from April to July 2018. This study followed the STROBE checklist form: Cross –sectional studies.

**Results:**

Overall, 88.6% (n=70/79) of participants knew about the M-CHW three home visits scheduled during pregnancy, 73.4% (n=58/79) about the three postnatal home visits after birth when the weight was normal, and 64.6% (n=51/79) about the five PNC home visits for low birth weights. Most (97.5%, n=77/79) knew that the mother and newborn should be screened during the same M-CHW home visits, and 87.2% (n= 68/79) were aware of the seven postnatal core competencies of delivering key maternal and newborn interventions during PNC home visits.

**Conclusions:**

There were varying levels of knowledge among the HB-MNHCP staff, indicating the need for ongoing monitoring and training to ensure that the correct information is provided to the mothers throughout the antenatal and postnatal periods. While most of the M-CHWs appear to have had the correct knowledge, their executing of some activities needs to be monitored to ensure that they provide the required services, as this is an important step in lowering the maternal and infant mortality and enabling Rwanda to meet its SDG 3. Home visits by the M-CHWs could increase referrals and reduce maternal and newborn mortality.

**Supplementary Information:**

The online version contains supplementary material available at 10.1186/s12884-022-04435-2.

## Background

Maternal mortality is defined as the death of a woman while pregnant or within 42 days of termination of pregnancy, irrespective of the duration and the site of pregnancy, from any cause related to or aggravated by the pregnancy or its management, but not from accidental or incidental causes [1], while neonatal deaths are those occurring within the first 28 days of life [2]. Approximately 125,000 women and 870,000 newborns die in the first week after birth every year in Africa due to the lack of adequate health care coverage [3]. Approximately 34% of maternal deaths that occur during the postnatal period on the continent have been attributed to hemorrhage, with complications such as infection and sepsis being responsible for 10% [3].

The large number of deaths of mothers with newborn babies, and of newborn babies, have resulted in continuing efforts in Africa to reduce them. The successes and limitations of these efforts are usually assessed in relation to the neonatal and maternal mortality rate. There were 47 neonatal deaths per 1000 live birth in Africa in 2019, the leading cause being preterm birth complications [4, 5]. In sub-Saharan (SSA), the annual newborn mortality is approximately 1.2 million deaths in the first 28 days of life [6]. While a number of countries in SSA has achieved substantive progress in reducing maternal and neonatal mortality rates, the numbers remain high, with marked variation between them. Many are not close to achieving the Sustainable Development Goal 3 (SDGs 3) targets of less than 70 maternal mortalities per 100,000 live births and less than 12 neonatal deaths per 1,000 live births by 2030 [7].

A number of other developing countries have made use of community health workers (CHW) to assist with health issues in general, and the maternal and neonate programmes in particular [8]. The roles and activities of CHWs are diverse, having been adapted within and across countries and programmes [8]. While in some cases the CHWs perform a wide range of tasks that can be preventive, curative and/or developmental, in other cases they are appointed for very specific interventions [8]. Of 86 articles discussing the use of specialized CHWs in developing countries, 30 focused on maternal and child health (including reproductive health and family planning), 19 on TB treatment, nine on malaria control, eight on acute respiratory infections (ARIs), seven on HIV/AIDS and 13 on other intervention areas [8].

Rwanda has recorded considerable progress in reducing maternal and neonatal mortality, although the rates remain higher than the SDGs 3 targets (less than 70 maternal mortalities per 100,000 live births, less than 12 neonatal deaths per 1,000 live births). The maternal mortality ratio decreased from 750 per 100,000 live births in 2005 to 210 in 2015, and neonatal mortality decreased from 37/1000 live births in 2005 to 20 in 2014/2015 [9–12]. Country level health statistics also usually analyses these rates in terms of mortalities per 1,000 live births that occur within 24 hours, seven and 28 days of birth to help indicate where particular challenges lie to prevent the deaths. Further improvements were achieved in 2012 due to the Rwandan Ministry of Health guidelines provided in the 2008 National Child Health Policy to accelerate the reduction of maternal and infant mortality [12, 13]. Rwanda supports maternal and newborn health interventions, for example, by ensuring that relevant policies, strategies and guidelines are in place, advising delivery at health facilities and recommending the use of modern contraception methods. In a study of 174 neonate deaths, 31% of infant and 5% of mothers’ deaths occurred at home [6, 10, 14].

A study also found that newborn mortality during the period of postnatal care accounted for approximately one-third of all child mortality, while 57% of women and 81% of newborns did not receive postnatal care services [6]. The study reported that the majority of neonatal deaths that happened within 48 hours of delivery could have been prevented [6]. This study is part of a larger study to critically analyse the implementation process of the maternal and newborn health care program in selected District Hospitals in Rwanda. This study therefore aimed to establish the knowledge of the health providers, providing HB-MNHC services as part of their antenatal, delivery and postnatal care program, specifically about the maternal community health workers (M-CHWs) services. In the absence of this information, it is not possible to know who among those involved in providing pregnant women, mothers and neonates with care and advice in the program are giving them the correct information about the services they can expect from the M-CHWs. The intention was to establish the knowledge of the different groups to see who in the system might not know what the M-CHWs do that could affect patients morbidity and mortality.

## Methods

### Study design and setting

In this study, a cross-sectional descriptive study design was used to establish the knowledge of health providers regarding their maternal and newborn health care program components of the HB-MNCP performed by M-CHWs [15]. This entailed included a cross-section of participants and using quantitative methods to collect data from the health workers who provide maternal and neonatal services in three districts that were purposively chosen from 30 in the three provinces. In each district, one district hospitals was purposively selected, with two of their associated health facilities being purposively selected (6 health facilities, 3 district Hospitals) (Table 1), one each in urban, peri-urban and rural areas. The post-positivists paradigm hold a determinist philosophy in which causes (probably) determine effects or outcomes [16]. The STROBE guidelines for reporting observational studies were included in reports of cross –sectional study checklist form were used to present the manuscript (See Supplementary file 1).

**Table 1:** District hospitals and health facilities included in the study

Province	District Hospital	Status	Health Facilities	M-CHWs	
				Total	30%
Kigali City	Muhima	Urban	Muhima	34	10
			Butamwa	44	13
Eastern	Nyamata	Peri-urban	Nyamata	35	10
			Mayange	35	11
Western	Kibogora	Rural	Kibogora	26	8
			Nyamasheke	50	15

The study population were all health providers (M-CHWs, nurses, midwives, nurse managers, data managers, health facility MCHWs supervisors and District hospitals M-CHWs supervisors) who were involved in managing, implementing and providing HB-MNHC services at various levels of services in the 30 districts in the three provinces, from which three districts and six health facilities were selected for inclusion as the study sample. Non-probability, convenience sampling was used to identify the study sample of 100 individuals to participate in the study.

The health care professional and data manager in charge of the maternal and newborn health care program were purposively selected from each health facility according to their availability during working hours, and consisted of one nurse manager, one M-CHW supervisor and one data manager. In the maternity ward, the nurses and midwives were included if they responded to the invitation to participate. At the district level hospital, the M-CHW supervisors were purposively selected due to their roles and responsibilities in the program, as well as 30% (*n*=67) of the 224 M-CHWs (Table 1).

Health providers who met the following criteria were included:

- 67 M-CHW: at least three years’ experience.
- 12 health facility based nurse and midwives in antenatal care, maternity ward and vaccination services: at least three years’ experience.
- 6 Health facility based M-CHW supervisors: at least three years’ experience.
- 6 Health facility based nurse manager: at least six months experience.
- 6 Health facility based data manager: at least six months experience.
- 3 District Hospital based M-CHW supervisors: at least three years’ experience.

The questionnaire was based on the literature that was reviewed, with questions being chosen from data collection instruments that had been pre-tested in Kenya, Indian, Malawi and the Philippines [13]. A pilot study was conducted using participants who were not included in the final study to test the data collection instruments for internal validation and reliability to ensure that the collection of appropriate data, with feedback being also used to adapt the questions.

The instruments were reviewed and refined before the main study was conducted and were modified to improve their clarity for a better understandable by the readers. The response rates was 85% of participants. with the Cronbach’s Alpha being .76, which has acceptable internal consistencies of above the 0.70, as indicated by Polite and Beck (2012) as being acceptable [17].

### Data collection

The data were collected by the researcher from April - July 2018, with appointments being made with the health facility and district hospital staff, while the M-CHWs were given the questionnaires at their monthly meeting and requested to complete it in the presence of the researcher. The concept of knowledge was selected as it implies a formal information transfer process, that specific details were provided during training/induction and assumed to have been acquired by the recipients with the intention that it be translated into action or transferred to patients.

The structured, close-ended questionnaire consisted of two sections, the first relating to demographic details (age, gender, education level, designation) to contextualize their results (Appendix, Supplementary file 1, point 1). The second section consisted of questions designed to obtain information regarding their knowledge about four components of the MCHWs role in the HB-MNCP: 1. the frequency of ANC care home visits; 2. the PNC visits for normal and low birth weight neonates; 3. screening and intervention home visits care for the mother and newborn, and 4. the seven core postnatal care competencies required by the M-CHWs to deliver key maternal and newborn interventions (Appendix, Supplementary file 1, point 2).

### Data analysis

The quantitative data were analysed using the statistical package for Social Sciences (SPSS 25.0), and entailed a descriptive analysis to summarise the findings, followed by statistical analysis using a chi-square test, with a P value < 0.05 being regarded as statistically significant. Chi-square and Fisher exact tests were performed to establish the association between the demographics variables and perceived frequency of home visits, antenatal care, postnatal care (newborns with normal and low weight), newborn and mothers screening, and maternal and newborn interventions conducted during the home visits. Only the findings showing statistically significantly difference are presented in this study.

### Ethical considerations

Approval for the study was obtained from Biomedical Research Ethics Committee (BREC) at the University of KwaZulu-Natal (BREC Ref No: BE029/18), and the Rwanda National Ethics committee (RNEC) (No.182/RNEC/2018). The mayors of the Bugesera, Nyamasheke, Nyarugenge Districts gave permission to conduct the study. Standard ethical considerations of anonymity and confidentiality were observed, as well as obtaining informed consent from the participants.

## Results

A summary of the demographic details is followed by the results of the four components of the MCHWs role in the HB-MNCP and the associated Chi-square and Fisher exact tests to establish any association between them, with only the statistically significant results being presented.

### Demographic details

The staff at each of the six health facility sites consisted of those working in the relevant program, with the numbers of those included in each category being indicated (Table 2). Of the 67 MCHWs who initially agreed to participate, 52 signed informed consent and completed the questionnaires. The facility based health providers were purposively selected from each facility according to their availability during working hours. Table 2 provides their demographic details, with most (65.8%) being M-CHWs, female (91.1%), aged 35-39 (55.1%) and with more than 4 years working experience (91.1%).

**Table 2:** Participants demographic characteristics (*N*=79)

Variables	Characteristic	n (%)
Designation	MCHW (67 invited)	52 (65.8)
	Health facility nurses and midwives (12 invited)	7 (8.9)
	Health facility nurse manager (6 invited)	6 (7.6)
	Health facility M-CHWs supervisor (6 invited)	6 (7.6)
	Health facility data manager (6 invited)	6 (7.6)
	District hospital M-CHW supervisor (3 invited)	2 (2.5)
Gender	Males	7 (8.9)
	Female	72 (91.1)
Settings	Rural areas	32 (40.5)
	Peri-urban	30 (38.0)
	Urban	17 (21.5)
Age group	25-29	11 (14.1)
	30-34	18 (23.1)
	35-39	43 (55.1)
	>=40	6 (7.7)
Years of work experiences	<=1	4 (5.1)
	1-3 years	3 (3.8)
	>= 4 years	71 (91.1)
Education	No formal education	2 (2.5)
	Primary school	42 (53.2)
	Secondary School	17 ( 21.5)
	University	18 ( 22.8)

#### Knowledge about the frequency of ANC care home visits

The majority of participants (n=78, 98.7%) knew that the first visit home done by M-CHWs took place as soon as a pregnancy is confirmed, while 88.6% (*n*=70/79) knew of the recommended frequency of three ANC home visits, which was statistically significant for gender, with females (*n*=68, 94.4%) being more aware than the males, and 100% (*n*=6) of the >= 40-year age group being aware compare to other age groups (F= 14.072, p< .045: two-sided). Regarding the designation, 100% (n=52) of the M-CHWs were aware of three visits compare to other designations. The majority with no formal (*n*=2, 100%) and primary education (*n*=42, 100%) were aware of three home visits compared to those with secondary and university qualification, the differences being statistically significant (F=24.210, *p*< .001: two-sided). The MCHWs (*n*=52, 100%) were aware of three home visits for pregnant women, compared to nurses and midwives (Table 3).

**Table 3:** Knowledge of frequency of MCHWs antenatal care home visits

**Antenatal Home Visit by ASMs**	**Yes**	**No**
	**n (%)**	**n (%)**
1. Once pregnancy is confirmed	78 (98.7)	1 (1.3)
2. At 5 – 6 months	70 (88.6)	9 (11.4)
3. At 8 - 9 months	72 (91.1)	7 (8.9)
**Overall knowledge of frequency of ASMs antenatal care home visits**	**N**	**%**
1. First ANC home visit	6	7.6
2. Second ANC home visit	2	2.5
3. Third ANC home visit	70	88.6

**Variables**	**Characteristic**	**N (%)**	**Value**	**P Value**
Gender	Males (n=7)	2 (28.6)	22.251	.000
	Female (n=72)	68 (94.4)		
Age group	25-29 (n=11)	7 (63.6)	14.072	.045
	30-34 (n=18)	15 (83.3)		
	35-39 (n=43)	41 (95.3)		
	>=40 (n=6)	6 (100)		
Designation	MCHW (n=52)	52 (100)	41.861	.000
	Health facility nurses and midwives (n=7)	5 (71.4)		
	Health facility nurse manager (n=6)	4 (66.7)		
	Health facility M-CHWS supervisor (n=6)	5 (83.3)		
	Health facility data manager (n=6)	3 (50)		
	District hospital-based M-CHW supervisor (n=2)	1 (50)		
Education	No formal education (n=2)	2 (100)	24.210	.001
	Primary school (n=42)	42 (100)		
	Secondary school (n=17)	16 (94.1)		
	University (n=18)	10 (55.6)		

#### Knowledge about PNC visits for normal and low birth weight neonates

Table 4 details the health providers’ knowledge about the frequency of postnatal visits required by M-CHWs after discharge from the health facility when birth weight is normal, and indicated that 73.4% (*n*=58/79) knew about all three home visits. The Chi-square and Ficher exact tests were performed to establish the association between knowledge about the frequency of postnatal care home visits by M-CHWs if birth weight is normal and participants’ characteristics. Females (*n*=56, 77.8%) were better informed than males (F= 9.912, *p*< .019: two-sided) and the majority (*n*=26, 86.7%) from semi-urban areas were more aware of three visits compared to those from rural and urban settings (F= 15.604, *p*<.003: two-sided). The majority (*n*=43, 82.7%) of M-CHWs knew the three visits compared to others designation (F= 25.987, *p*< .012: two-sided). Regarding level of education, most of those who completed primary school (n=35, 83.3%) knew about the three visits compare to those with high qualifications (F= 16.599, *p*< .023: two-sided) (Table 4).

**Table 4:** Knowledge of frequency of MCHWs PNC home visits: Normal birth weight

**Visit days**	**Yes**	**No**
	**N (%)**	**N (%)**
1. Within 24 hrs after discharge	74 (93.7)	5 (6.3)
2. Days 5-7 after delivery	62 (78.5)	17 (21.5)
3. Day 28 after delivery	72 (91.1)	7 (8.9)
**Overall knowledge of frequency of ASMs PNC home visits : Normal weight**	**N**	**%**
1. First PNC home visit	4	5.1
2. Second PNC home visit	12	19
3.Third PNC home visits	58	73.4

**Variables**	**Characteristic**	**N (%)**	**Value**	***P*** **Value**
Gender	Males (n=7)	2 (28.6)	9.912	.019
	Female (n=72)	56 (78.8)		
Settings	Rural areas (n=32)	23 (71.9)	15.604	.003
	Peri-urban (n=30)	26 (86.7)		
	Urban (n=17)	9 (52.9)		
Designation	MCHWs (n=52)	43 (82.7)	25.987	.012
	Health facility nurses and midwives (n=7)	4 (57.1)		
	Health facility Nurse manager (n=6)	4 (66.7)		
	Health facility M-CHWs supervisor (n=6)	4 (66.7)		
	Health facility Data manager (n=6)	3 (50)		
	District hospital-based M-CHWs supervisor (n=2)	0 (0.0)		
Education	No formal education (n=2)	1 (50)	29.165	.001
	Primary school (n=42)	35 (83.3)		
	Secondary School (n=17)	13 (76)		
	University (n=18)	9 (50)		

Regarding the first postnatal visit by M-CHWs after discharge from the health facility when birth weight is low, the majority (*n*=72, 91.1%) knew that postnatal care home visit 1 occurs when the mother arrives home or within 24 for home delivery. Table 5 details the varying levels of knowledge about the frequency of home visits required for low birth weight infants.

**Table 5:** Knowledge of frequency of M-CHWs PNC home visits: low birth weight (LBW)

**Visit days**	**Yes**	**No**
	**N (%)**	**N (%)**
1. When the mother arrives home or within 24 hrs after birth	72 (91.1)	7 (8.9)
2. Day 5 after delivery	56 (70.9)	23 (29.1)
3. Day 7 after delivery	70 (88.6)	9 (11.4)
4. Day 14 after delivery	69 (87.9)	10 (12.7)
5. Day 28 after delivery	70 (88.6)	9 (11.4)
**Overall knowledge of frequency of MCHWs PNC home visits : LBW**	**N**	**%**
1. First PNC home visit	4	5.1
2. Second PNC home visit	3	3.8
3. Third PNC home visit	4	5.1
4. Fourth PNC Home visit	15	19
5. Fifth PNC home visit	51	64.6

**Variables**	**Characteristic**	**n (%)**	**Value**	**P value**
Gender	Males (n=7)	2 (28.6)	18.089	.001
	Female (n=72)	49 ( 68.1		
Settings	Rural areas (n=32)	19 (59.4)	33.692	.000
	Peri-urban (n=30)	26 (86.7)		
	Urban (n=17)	6 (35)		
Age group	25-29 (n=11)	6(54.5)	20.603	.046
	30-34 (n=18)	8 (44.4)		
	35-39 (n=43)	30 (69.8)		
	>=40 (n=6)	6 (100)		
Designation	MCHWs (n=52)	39 (75)	49.506	.000
	Health facility nurse and midwives (n=7)	3 (42.9)		
	Health facility nurse manager (n=6)	2(33.3)		
	Health facility M-CHWs supervisors (n=6)	4 (66.7)		
	Data manager (n=6)	3 (50)		
	District hospital–based M-CHWs supervisor (n=2)	0 (00)		
Education	No formal education (n=2)	1 (50)	26.393	.007
	Primary school (n=42)	31 (73.8)		
	Secondary School (n=17)	12 (70)		
	University (n=18)	7 (38.9)		

The majority (*n*=26, 86.7%) from semi-urban areas were better informed than those from other settings. Respondents aged >= 40 years (*n*=6, 100%) were also more informed than others group age, the difference being statistically significant (F= 20.603, *p*< .046: two-sided). The females (n=49, 68.1%) were more knowledgeable than the males, the difference being statistically significant (F= 18.089, *p*< .001: two-sided). More M-CHWs (75%) knew about the five visits compared to others designations, while most of those who completed primary school (*n*=31, 73.8%) knew compare to those with a high qualification (F= 26.393, *p*< .007: two-sided) (Table 5).

#### Knowledge about screening and intervention home visits care for the mother and newborn

Regarding the screening interventions provided by the M-CHWs for mothers and newborns at their home visits, 97.5% (n=77) knew that this must occur (Table 6).

**Table 6:** Knowledge about screening of mothers and newborns

	N	Yes		No		Chi-square		
		n	%	n	%	Value	df	***P*** Value
Mother and newborn are screened at the same time	79	77	97.5	2	2.5	71.203	1	.000

#### Knowledge about the seven core postnatal care competencies required by the MCHWs to deliver key maternal and newborn interventions

Regarding the core PNC competencies required to deliver key maternal and newborn interventions, over 90% of the respondents know about at least some of them, with 87.2% (*n*= 68/79) being aware of all seven postnatal (Table 6). The urban respondents (*n*=17, 100%) were more knowledgeable than those from peri-urban and rural areas, the difference being statistically significant (F= 14.718, *p*< .047: two-sided) (Table 6). In terms of the designation, all (*n*=6, 100%) the nurse managers were more knowledgeable than the other categories, the difference being statistically significant (F= 53.531, *p*< .018: two-sided) (Table 7).

**Table 7:** Knowledge of 7 core competencies of MCHWs PNC home visits

**Maternal and newborn interventions**	**Yes**	**No**
	**N (%)**	**N (%)**
Newborn care early/exclusive breastfeeding, warmth, hygiene	75 (94.9)	4 (5.1)
Care for mother nutrition and family planning	73 (92.4)	6 (7.6)
Care seeking mother or mother and newborn	75 (94.9)	4 (5.1)
Identify danger sign in mother plus referral	75 (96.2)	3 (3.8)
Identify danger sign in newborn plus referral	76 (96.2)	3 (3.8)
Support for breastfeeding	74 (93.7)	5 (6.3)
Feeding, skin-to-skin contact	75 (94.9)	4 (5.1)
**Overall knowledge of core competencies of MCHWs PNC home visits**	**N**	**%**
One core competencies	1	1.3
Two core competencies	1	1.3
Three core competencies	1	1.3
Four core competencies	1	1.3
Five core competencies	1	1.3
Six core competencies	5	6.4
Seven core competencies	68	87.2

**Variables**	**Characteristic**	**N (%)**	**Value**	**P. Value**
Settings	Rural areas (n=32)	23 (71.9)	14.718	.047
	Peri-urban (n=30)	28 (96.6)		
	Urban areas (n=17)	17 (100)		
Designation	M-CHWs (n=52)	47 ( 92.2)	53.531	.018
	Health facility nurses and midwives (n=7)	5 (71.4)		
	Health facility nurse manager (n=6)	6 (100)		
	Health facility M-CHWs supervisor (n=6)	5 (83.3)		
	Health facility Data manager (n=6)	4 (66.7)		
	District hospital-based M-CHW supervisor s (n=2)	1 (50)		

## Discussion

The study aimed to establish health provider’s knowledge about home–based maternal and neonatal health care package guidelines to identify possible areas that may be affecting the promotion and uptake of related services. This included exploring the association between the participants’ demographic characteristics and their knowledge about maternal community health workers home visit, with issues such as frequency, during of antenatal and postnatal periods, when the birth weight is both normal and low, and the screening and interventions of the mothers and newborns.

### Demographic characteristics

The majority of those who participated in this study were female (91.1%) M-CHWs (65.8%), which is similar to a study in Uganda [18], and meets the Rwandan policy criteria for selecting female M-CHW who were involved in maternal and newborn health [12, 13, 19, 20]. Engaging male M-CHWs to provide services can be beneficial when there are insufficient female volunteers [18]. Another Rwandan study reported that 14% of M-CHWs were males, their responsibilities including maternal and newborn health [21]. However, the inclusion of males as M-CHWs needs to be provided for in the Rwanda National Community Policy, which currently only makes provision for female service providers.

The predominance of females is in line with the HB-MNHCP selection criteria of females aged 20 – 50 who can read and write, as per the National Community Health Policy [20], and as indicated in other studies [20–22]. The fact that men have been employed could indicate the absence of female applicants, and the need to fill the posts to ensure that services are provided to reduce maternal and neonatal mortality and morbidity.

The majority of participants had completed primary school, which corresponds to most being MCHWs, the criteria for their selection being the ability to read and write [23]. Previous studies indicated a shortage of health professionals in many public sector facilities, specifically in rural areas, which affects training, supervision and monitoring of the MNH care program. These shortages were the reason that the Ministry of Health decided to recruit M-CHWs, as there were insufficient opportunities for rural communities in particular to access information and support services, which impacted on the health outcomes of mothers and newborns. The large number of M-CHWs provide an essential service as a first level of accessible care and justified their inclusion in this study [22, 24, 25]. A study found that while the M-CHWs may not have the capacity to provide a wide range of health services, they can influence and encourage health services utilization [21]. Community-based newborn programs that include home visits for care can improve neonatal survival in the first week of life in collaboration with facility-based PNC services [26]. The differences between the participants’ demographic variables and their awareness about the M-CHWs areas of work were statistically significant for: 1. frequency of antenatal home visits, 2. postnatal care visit for with normal and low birth weight, 3. screening and intervention home visits care of mothers and newborns.

#### Knowledge about the frequency of ANC care home visits

As indicated in Table 3, there should be three scheduled M-CHW visits during pregnancy, and the women should be encouraged to attend health facility during this time and for delivery. Providing the women with relevant information is anticipated to improve ANC utilization, specifically in areas with limited access to health services, and highlights the need for community and household mobilization to be done, as per the WHO's recommended home-based care strategy [27]. Ensuring the accessibility and utilization of health service is a prerequisite for effective antenatal care and as a delivery platform for other health services. In addition, the timing of ANC visit and organization of health services are important. For example, in rural Zimbabwe and Malawi, follow-up of mothers and infants during ANC and PNC visits was affected by the centralization of some services to higher levels of care. To address these challenges, measures were taken to extend ANC activities to lower level health facilities, strengthen community outreach programs and improve geographical access [28] .

A study in Rwanda reported that some M-CHWs only provided ANC within the first four months of pregnancy (63%), while 37% received four or more consultations, and 70% delivered in a health facility facilitated by a skilled health provider [21, 29]. The home visit frequency schedule during pregnancy needs to be conducted according to the country’s requirements, such as in Afghanistan, where four home visits are scheduled [30]. Such visits are important, as they provide opportunities for discussions around pregnancy-related issues, and enable reminders for the mothers to attend facility-based check-ups, these being necessary to identify any possible problems that need to be addressed at the delivery.

The older age group had better knowledge of the ANC home visits scheduled than the younger participants, possibly due to their manager and supervisory positions, or having had years of experience in the programme that indicated the value of such a schedule. This is contrary to a study in 2016, which reported that M-CHWs 50 years old and older were difficult to retain and did not necessarily apply the knowledge and skills they acquired [23].

A Liverpool School of Tropical Medicine survey of M-CHWs in Rwanda found that a similar number had no formal (2.5%) and primary school (53.2%) education. As in the current study, they found that M-CHWs were more aware of the ANC home visits schedule than the nurses and midwives. The younger and more qualified participants with secondary school and university qualification may be health facility-based, their responsibilities being in the wards, where staff shortages result in them focusing on the issues at hand rather than on externally provided services, such as training and supervision of M-CHWs [23, 31].

Should the three ANC home visits be implemented as required, the pregnant women could be motivated to attend the health facility for the recommended four visits, with a similar study showing that conducting the first standard ANC visit during the first trimester and the fourth visit influenced maternal and neonatal outcomes [6]. In 2016, the national average for ANC visits to a health facility /or by a healthcare worker was 44% for the first and 37% for the fourth visit. The author highlighted that the delay in attending the first ANC standard visit during the first trimester also leads to a low attendance of the remaining four ANC standard visits. Some district hospitals, which compiled the report for their health facility catchment areas, reported that the average number of visits for ANC services were low at under 44% and others higher than 60% [32]. The district hospital report showed that the number of first and fourth ANC standard health facility visits were very similar, irrespective of percentage [32] .

This highlights the importance of the three ANC home visits by the M-CHWs to remind and encourage the women to attend their appointments at the health facilities. The WHO guideline recommends eight scheduled ANC attendance of pregnant women at health facility, the first contact during the 12^th^ weeks of gestation, with following contacts taking place at 20, 26, 30, 36, 38 and 40 weeks to ensure a good pregnancy outcome [33, 34]. The study revealed a knowledge gap between the required three ANC home visits by the M-CHWs during pregnancy, as indicated by the number of pregnant women attending health facilities. In the context of Rwanda, the number of pregnancy women who attended four or more consultations was low (44 %) [33]. This suggests the need to revise the ANC home visits scheduled by M-CHWs during pregnancy and to follow-up its implementation by the Rwanda Ministry of Health.

#### Knowledge about PNC visits for normal and low birth weight neonates

Overall participants knew about the M-CHWs three PNC home visits schedule after birth when the weight was normal, and 64.6% about the five visits for low birth weights. Adhering to the scheduled visits for normal and low weight babies could contribute to reducing maternal and newborn deaths within 48 hours following delivery, especially after home delivery [35]. The same authors found that the M-CHWs accompanied 64% of mother and baby after home delivery to the health facility. They also performed postnatal home visit 1 for 39% of the mother/newborn, and visit 2 for 36% [35]. The Rwanda Demographic and Health survey, 2014/15, reported that only 19% of newborns received PNC in the first two days after birth, indicating that the M-CHWs may not be conducting the visits, despite indicating that they know that they should be doing them [11].

The nurses and midwives did not know about the three home visits if the birth weight is normal, indicating the need for them to be better informed about the HB-MNHCP during their training and continuing professional development (CPD) opportunities [20–22]. These health care providers have contact with the mothers at the health facilities during their antenatal visits and at the time of delivery, and need to motivate them to call on the M-CHWs to provide PNC support visits. The district hospital M-CHWs supervisor reported that the health facility workers (nurses, midwives) often do not give mothers referrals forms after facility deliveries or are not aware of the importance of ensuring community follow-up by the M-CHWs [13]. This indicates the need for training to be provided to the nurses and midwives who deal with the mothers and neonates to provide them with referral letters to ensure that the received the necessary home visits.

Regarding the frequency of home visits if birth weight is normal and low, in general, the M-CHWs were better informed than other health providers (nurses and midwives). This is evidenced by another study conducted in the same areas, which found that the PNC visits by M-CHWs were appreciated due to the shortage of other health care cadre who would have done such visits [6]. It reported that when other health providers were required to perform community-based PNC services they considered it to be an additional duty due to their high health facility-based workload [6]. Another author indicated that there were insufficient ANC providers at health facilities, and that the information being given to pregnant women attending such services was inadequate in terms of their identifying danger signs of pregnancy, taking the recommended medicines and undergoing tests [34] .

The significance PNC home visits for mothers and newborns are regarded as being during the first six weeks after childbirth, which is the period of increased care needs [6]. In 2013, the WHO added four postnatal visits guidelines for covering the danger period of mother and baby in the six weeks after birth, which was included in the 2016 Rwandan services. The four PNC components includes physical and medical examinations as well as counseling on different topic for the mother and baby [6].

#### Knowledge about screening and intervention home visits care for the mother and newborn

The M-CHWs home visits, as indicated in the guidelines [13]. PNC screening home visits can reduce mortality and morbidity by identifying problems, assisting with decisions to improved infant care, and advising on referrals for mothers and newborns [6]. Such screening measures by the M-CHWs can assist in reducing maternal and neonate mortality, as mothers may not appreciate the severity of the infant’s condition, and only seek health care services when it is too late. Timeous access to care, for both the mother and the neonate, is essential if services are going to be accessed that make the difference between life and death, with 43% of mothers and 19% of newborns receiving a postnatal checkups [6].

#### Knowledge about the seven core postnatal care competencies required by the MCHWs to deliver key maternal and newborn interventions

Most participants were aware of the seven core postnatal care competencies in order to deliver key maternal and newborn interventions, which may have contributed to the reduction in maternal and newborn mortality after health workers were trained in their application after 2010. A previous study in the same area revealed that more than 50% of the M-CHWs were confident in their ability to provide PNC services [23], this low number being of concern, as they provide such an essential service to many women who may otherwise not have access to pregnancy-related advice.

There were statistically significant differences in the current study between the participants’ knowledge about the seven core postnatal care competencies from urban, peri-urban and rural areas. All the nurse managers were better informed than the health facilities M-CHWs supervisors, nurses and midwives, data managers and district hospital-based M-CHWs supervisor about the seven core competencies. The nurse managers may be more knowledgeable about intervention care, as indicated in another study, as they have received more extensive training on the initiative, and are responsible for training and advising other staff members under their supervision [36].

The study may have been affected by a number of limitations, these including that only a few of some categories of designations participated and only six facilities were included. In addition, it is not possible to establish to what extent what the M-CHWs know they should do translates into them actually providing those services and in the manner required. As a quantitative study, it did not establish the details about what was known about each component of the study. However, it does indicate the gaps in knowledge at the various levels of care about what services the M-CHWs provide for the women who need ANC and PNC services. It indicates the need to undertake studies among the community-based women recipients of the M-CHW care to explore the information they receive from the M-CHWs and the other role players in the pathway of care with whom they interact while receiving services.

## Conclusion

There were varying levels of knowledge among the HB-MNHCP staff, indicating the need for ongoing monitoring and training to ensure that the correct information is provided to the mothers throughout the antenatal and postnatal periods. While most of the M-CHWs appear to have had the correct knowledge, their executing of some activities needs to be monitored to ensure that they provide the required services, as this is an important step in lowering the maternal and infant mortality and enabling Rwanda to meet its SDG 3. Home visits by the M-CHWs could increase referrals and reduce maternal and newborn mortality.

Establishing the various health care provides knowledge about what maternal and neonate services are offered as part of the community-based HB-MNHCP by M-CHWs is important, as its implementation needs the support of all those who engage with the care recipients. The results suggest that the M-CHWs are aware of their responsibilities in terms of ANC and PNC, and are able to provide mothers and community members with information that will encourage the women to attend the health facilities for more specialized care. The responses from the supervisors and data managers suggests that they are provided with the resources to enable the referrals to happen, but that the nurses and midwives may not provide them with letters to take back to the MCHWs. The M-CHWs were largely aware of the seven core competencies, the results collectively suggesting that while the systems are in place to enable information to be dispensed and services provided, that a number of gaps need to be addressed if the maternal and neonate mortality and morbidity is to be reduce to the SDGs requirements.

Ongoing monitoring and evaluation is therefore required to establish what problems or challenges are occurring in getting women to attend ANC and PNC services, and to ensure that the strategies and plans remain relevant and are implement appropriately. This includes engaging males in all positions of the program, including as M-CHWs, as done elsewhere, specifically where the absence of women result in a gap in services, which could affect the programs outcome. The M-CHWs, who are at the forefront of care, need to receive the necessary training and support to ensure that they have the knowledge and confidence to offer the essential support services to improve maternal and newborn survival for SDG goal 3 to be achieved.

## Supplementary Information

**Additional file 1.** Appendix Supplementary file 1

## Data Availability

The datasets used and/or analysed during the current study are available from the corresponding author on reasonable request.

## Abbreviations

ANC: Antenatal care
ASM: Animatrice Santé Maternelle
BF: Breast feeding
BREC: Biomedical Research Ethics Committee
CHWs: Community Health workers
CMWs: Community midwives
CPD: Continuing professional development
HB-MNHC: Home-Based Maternal and Neonatal Health Care
HB-MNHCP: Home-Based Maternal and Neonatal Health Care Package
HF: Health Facility
HMIS: Health Management Information System
LSTM: A Liverpool School of Tropical Medicine
MCH: Maternal child health
MCHIP: Maternal and Child Health Integrated Program
M-CHWs: Maternal community health workers
MNH: Maternal newborn health
MOH: Ministry of Health
PIP: Project Implementation Profile
PMI: Project Management Institute
PNC: Postnatal care
RBC: Rwanda Biomedical Center
RNEC: Rwanda National Ethics committee
SGDs: Sustainable Development Goals
SIScom: Systeme d’Information Sanitaire des Communautes/CHW information system
SPSS: Statistical package for Social Sciences
SSA: sub-Saharan Africa
UNICEF: United Nations International Children’s Emergency Fund
WHO: World Health Organization
