# Supplementary material for: Exploring health provider’s knowledge on the home-based maternal and neonatal health care package in Rwanda
Source: BMC Pregnancy Childbirth. 2022 Feb 7;22:107. doi: 10.1186/s12884-022-04435-2 (PMC8819836; doi:10.1186/s12884-022-04435-2)
Supplement: Supplementary file 1 — Additional file 1. Appendix Supplementary file 1 [file 12884_2022_4435_MOESM1_ESM.docx]

**Participants**

1. Maternal community health workers (MCHWs), health facility and district hospital based community supervisor, nurse managers, data managers, and staff working in maternity ward (**Participants)**

**Demographic data**

**Settings: PHC:** Rural areas: **1. ----** Peri-urban **2. ----** Urban 3. **----** District Hospital **……………..**

**Participant number: Date:**

| **Participants:** |  |  |
| --- | --- | --- |
| 1.Designation | MCHW | 1 |
|  | Health facility nurses and midwives | 2 |
|  | Health facility nurse manager | 3 |
|  | Health facility M-CHWs supervisor | 4 |
|  | Health facility data manager | 5 |
|  | District hospital M-CHW supervisor | 6 |
| 2.Gender | Males | 1 |
|  | Female | 2 |
| 3.Age group | 25-29 | 1 |
|  | 30-34 | 2 |
|  | 35-39 | 3 |
|  | >=40 | 4 |
| 4. Year of work experiences | <=1 | 1 |
|  | 1-3 years | 2 |
|  | >= 4 years | 3 |
| 5. Education | No formal education | 1 |
|  | Primary school | 2 |
|  | Secondary School | 3 |
|  | University | 4 |
|  |  |  |

1. **Knowledge about maternal and newborn health care program performed by MCHWs.**

| **Frequency of home visit by MCHWs** | | |  |
| --- | --- | --- | --- |
| 1.      Antenatal period | Three home visits |  |  |
|  | Pregnancy visit 1: as soon as pregnancy is confirmed | Yes | No |
|  | Pregnancy visit 2: Five-six months of pregnancy | Yes | No |
|  | Pregnancy vsit3:between eight –nine months of pregnancy | Yes | No |
|  |  |  |  |
| 2.     Postnatal home visit ( newborn with normal birth weight ). | Postnatal visit 1: With 24 hours after discharge from health facility or home delivery | Yes | No |
|  | Postnatal 2:between day5-7 after delivery | Yes | No |
|  | Postnatal visit 3: day 28 after delivery | Yes | No |
|  |  |  |  |
| 3.      Postnatal home visits ( newborn with low birth weight). | Postnatal visit 1: when the mother arrives at home or within 24 hours for a home delivery | Yes  Yes | No  No |
|  | Postnatal visit 2: day 5 after delivery | Yes | No |
|  | Postnatal visit 3: day 7 after delivery | Yes | No |
|  | Postnatal visit 4: day 14 after delivery | Yes | No |
|  | Postnatal visit 5: 28 days after delivery | Yes | No |
|  |  |  |  |
|  |  |  |  |
| 4.Mother and newborns are screened at the same time | Yes | 1 |  |
|  | No | 2 |  |
| 5. Maternal and newborn interventions conducted during the home visit | Promotion of newborn care ( early/exclusive BF, warmth hygiene); | Yes | No |
|  | Promotion of optimal care for mother ( nutrition& family planning); | Yes | No |
|  | Promotion of care- seeking or mother &newborn; | Yes | No |
|  | Identification of danger signs in mother +referral; | Yes | No |
|  | Identification of danger signs in newborn+ referral; | Yes | No |
|  | Support for breastfeeding; | Yes | No |
|  |  |  |  |
|  | Care of low birth weight infant (feeding, skin-to skin contact). | Yes | No |
|  |  |  |  |
|  |  |  |  |

1. **Strategies to ensure the compliance with guidelines of maternal and newborn health care program performed by MCHWs.**

| 1.Home visit by health care provider | 1day per week | Yes | No |
| --- | --- | --- | --- |
|  | 1 time by month | Yes | No |
|  | Other alternative:…….. | Yes | No |
| 2. How many delivery rooms do you have in your health facility? | At least 1 room | Yes | NO |
|  | 2 room | Yes | No |
|  | 3 Rooms | Yes | No |
| 3. Management of pre-term and low birth weight babies | Kangaroo mother care (KMC) | Yes | No |
|  | Refer sick newborns with danger signs | Yes | No |
| 4. Record keeping from the first ANC visit | Yes: | 1 |  |
|  | No. | 2 |  |
| 5.Refer process of a pregnant women | Referral note | 1 | Yes |
|  | Given referral note during discharge for continuation in Community | 2 | No |
| 6. Supervision and evaluation of PHC by district hospital based community supervisor | Weekly | 1 |  |
|  | Monthly | 2 |  |
|  | Quarterly | 3 |  |
|  | Annually | 4 |  |
|  | Not at all | 5 |  |

1. **Stakeholder’s perception of existing and extend of critical success of maternal and newborn health care program implementation**

| **Critical success of MNHC program** | **Strongly disagree** | **Disagree** | **Neither agree nor disagree** | **Agree** | **Strongly agree** |
| --- | --- | --- | --- | --- | --- |
| **1**.A written statement mission related to the maternal and newborn health care program, the goals have been explained to all personnel affected by the project |  |  |  |  |  |
| **2**.Manager understands the amount of resources (money, time, manpower, equipment, training) required to implement this program |  |  |  |  |  |
| **3**.Manager had a detailed plan including time schedules, crucial documents manpower requirement , equipment for performing the program |  |  |  |  |  |
| **4**. The mothers are kept informed of the program’s progress in terms of antenatal care visit, delivery, emergency obstetric care, management of sick newborn and PNC home visit situations. |  |  |  |  |  |
| **5**. Health Center has enough trained health providers to perform the program. |  |  |  |  |  |
| **6**.The RapidSMS-MCH system is being implemented and works well |  |  |  |  |  |
| **7**. Mother, MCHWs, health provider knows who to contact when problems or questions arise related to the program |  |  |  |  |  |
| **8**.Regular meetings to monitor program progress and improve the feedback to the program team |  |  |  |  |  |
| **9**.The program goals have been well defined and explained to health providers of the program team, MCHWs, mothers and their family groups affected by program work. |  |  |  |  |  |
| **10**.Manager addressed "problem areas" by discussing them with MCHWs, mothers, mothers-in-law and husband and identify a solution strategy |  |  |  |  |  |
